# Autonomic dysfunction as a possible cause of sudden cardiac death in swimming sports

**DOI:** 10.3389/fcvm.2024.1443214

**Published:** 2024-08-22

**Authors:** Jiri Dostal, Tereza Hybska, Khatuna Saganelidze, Radek Pudil, Josef Stasek

**Affiliations:** ^1^ 1st Department of Internal Medicine, Faculty of Medicine in Hradec Kralove, Charles University, Hradec Kralove, Czechia; ^2^ Center for Sports Medicine, Prague, Czechia; ^3^ New Vision University, Tbilisi, Georgia

**Keywords:** diving reflex, autonomic dysfunction, syncope, sudden cardiac death, bradycardia, hemodynamics

## Abstract

**Introduction:**

Human diving reflex is a well-studied phenomenon. However, very little is known about the possible relationship between augmented diving reflex and autonomic dysfunction.

**Methods:**

We retrospectively studied a group of four swimmers who underwent a diving reflex test as part of the examination due to symptoms related to autonomic dysfunction during swimming. The control group comprised 11 healthy swimmers with no history of these symptoms. A standardized diving reflex test was performed for each athlete in both groups. Hemodynamic profiles, including heart rate, stroke volume, and cardiac output, were recorded.

**Results:**

There were no statistically significant differences between the groups in any of the three parameters measured before the test. However, at the end of the test, each parameter (heart rate, stroke volume, and cardiac output) was significantly lower in the swimmers who presented with clinical symptoms related to autonomic dysfunction than in the control group.

**Conclusion:**

This observation could shed light on autonomic dysfunction as a possible cause of sudden cardiac death in swimming athletes. It also demonstrated that autonomic dysfunction is presented not only by decreased heart rate but also by stroke volume, causing a drop in cardiac output to the level of hemodynamic collapse.

## 1 Introduction

The mammalian diving reflex is defined as a set of physiological responses initiated upon immersion of the face in cold water. It is characterized by several physiological characteristics, such as slowing of the heart rate (HR), decreased cardiac output with peripheral vasoconstriction via sympathetic stimulation, increased mean arterial blood pressure (MABP), and contraction of the spleen (1). Sudden cardiac death in water sports has been previously studied with conflicting evidence of root causes (2, 3); however, no relation to possible autonomic dysregulation has been mentioned. More recently, the hypothesis that the mammalian diving reflex is the root cause of several catastrophic events, including sudden cardiac death and other severe pathologies, has been discussed, but without any clear evidence of attenuated results of the face water immersion test (4).

## 2 Aim of the study

The aim of this study was to identify the differences in hemodynamic response in individuals who were presented to the cold water face-immersion test (diving reflex) with clinical symptoms related to autonomic dysfunction in swimming and in control groups of healthy individuals. The hypothesis was that individuals who presented to test for clinical symptoms also demonstrated a hemodynamically augmented diving reflex with not only severe bradycardia, but other cardiovascular parameters as well. This observation may shed light on the possible root cause of sudden cardiac death during swimming.

## 3 Materials and methods

In this study, we retrospectively analyzed the data from diving tests performed in clinical practice. The indication for the test was either clinical or preventive. The clinical indication was in individuals who were referred to our clinic for health checks due to presyncope, syncope, and dizziness during swimming in water when training or competing. We did not include any divers or free divers with symptoms of shallow water blackout, as the pathogenesis is known and described elsewhere (5). Prevention-related indications included swimmers, divers, or free divers who requested the diving reflex test as part of their regular preparticipation screening and who were free of the symptoms described above. Data processing was approved by the Ethical Committee of the Center for Sports Medicine (code 01-2024). All participants signed an informed consent form before the face-immersion test.

### 3.1 Participants

A total of 11 healthy individuals with a background in water sports (swimming, artistic swimming, diving, or freediving) were included in the arm of preventive indication. None of them experienced any clinically relevant symptoms during swimming or diving. The patient group included four individuals with a history of clinical symptoms (including presyncope, loss of consciousness, loss or orientation, or blackouts, but not limited to) while swimming or during shallow water diving (artistic swimmers). All participants in both groups underwent full cardiac screening, including physical examination, resting ECG, and stress ECG, with normal results before the diving reflex test.

### 3.2 Protocol

A basin of cold water was prepared for face immersion in advance. The temperature was monitored to reach 7°C–10°C during the test. The basin was approximately 50 cm wide with a water depth of 10 cm to ensure immersion of the full face. The test was performed by a physician, nurse, or physician assistant to ensure appropriate reactions in case of any sudden cardiovascular issue. The participant was rested for 5 min seated, while connected to an ECG (Schiller AT104, Baar, Switzerland) and a non-invasive hemodynamic monitoring system (PhysioFlow Enduro, Menatec, Poissy, France). The participant then carefully lay prone with the head freely placed over the basin of cold water. The familiarization dry test took place immediately before the test and started with a few deep inhales and exhales with breath hold until exhaustion over the water without immersion. The test itself was guided by lab staff instructing the participant to immerse the whole face, with a special focus on immersing the chin and forehead to ensure that all three exits of trigeminal nerves were immersed in water. After a few deep inhales and exhales, the participant immersed their face in cold water until exhaustion. The pathological response was defined as a 50% reduction in HR and/or pauses longer than 3 s and/or the appearance of complex ventricular arrhythmias connected to the symptoms in the patient’s history. This definition is our interpretation of the results, based on the best clinical knowledge and published data from other centers performing the procedure. There is no general consensus and no official guidelines have been published to date.

### 3.3 Recording procedure

A 12-lead ECG (Schiller) with modified Mason–Likar leads and continuous recording was used. Data were displayed in the ECG proprietary software for analysis. The onset and end of breath holding were marked by markers on the ECG. Non-invasive hemodynamic monitoring was performed using impedance cardiography (PhysioFlow). The recorded data included the stroke volume, heart rate, and cardiac output. The detailed principles of this method have been described elsewhere (6). The immersion time was measured using a standard lab-grade stopwatch.

### 3.4 Statistical analysis

The Welch two-sample *t*-test (unequal variances *t*-test) was used because of the unequal numbers of participants in each arm. Statistical significance was set at *p* < 0.05. Data are expressed as mean ± SD.

## 4 Results

In total, 15 individuals participated in the study: six women (3/4 in the pathology group and 3/11 in the healthy group) and nine men. Of the 15 participants, 11 did not present any clinical evidence of a possible pathology. Of the 15 participants, 4 were present in the test for medical reasons, as mentioned earlier in the text. The mean age of the participants was 20 ± 4 and 17 ± 3 years in the healthy group and pathology group, respectively.

There was no statistically significant difference in heart rate between the groups at baseline (*p* = 0.44). The mean baseline heart rate was 83 ± 16 and 90 ± 14 bpm in healthy individuals and patients, respectively. The stroke volume at baseline was also not significantly different between the groups (*p* = 0.72). The mean stroke volume at baseline was 92 ± 21 and 89 ± 11 ml in healthy individuals and patients, respectively. Thus, the cardiac output did not differ between the groups at the beginning of the test (*p* = 0.60). Healthy individuals had a mean cardiac output of 7.49 ± 1.6 L/min, while it was 7.98 ± 1.43 L/min in patients.

The end-test minimal HR was significantly lower in the patient group (*p* < 0.0005). The minimum heart rate in the patient group was 27 ± 6 bpm and the minimum heart rate in healthy individuals was 51 ± 13 bpm.

Stroke volume at the end of the test was significantly lower in the patient group (*p* < 0.01). The minimal stroke volume in the patient group was 49 ± 9 ml, whereas the minimal stroke volume in healthy individuals was 68 ± 14 ml.

It has been altogether demonstrated that cardiac output was significantly lower in the patient group (*p* < 0.001). The minimal cardiac output in the patient group was 1.32 ± 0.38 L/min, while the minimal cardiac output in healthy individuals was 3.36 ± 0.73 L/min.

The mean decrease in heart rate in healthy individuals was 39% ± 6%, while that in patients dropped by 70% ± 6%, with a statistically significant difference (*p* < 0.001). The same trend was observed for stroke volume, where the decline in healthy individuals was 25% ± 8%, whereas in the patient group, the drop was 45% ± 11%. The results were statistically significant (*p* < 0.02). Cardiac output declined by 54% ± 9% in healthy individuals and by 83% ± 4% in patients. The result was also statistically significant (*p* < 0.001).

All measured hemodynamic parameters demonstrated statistically significant differences between the groups. All four patients demonstrated clinically identical symptoms while swimming or diving, while none of the healthy individuals experienced similar symptoms.

**Figure 1:**
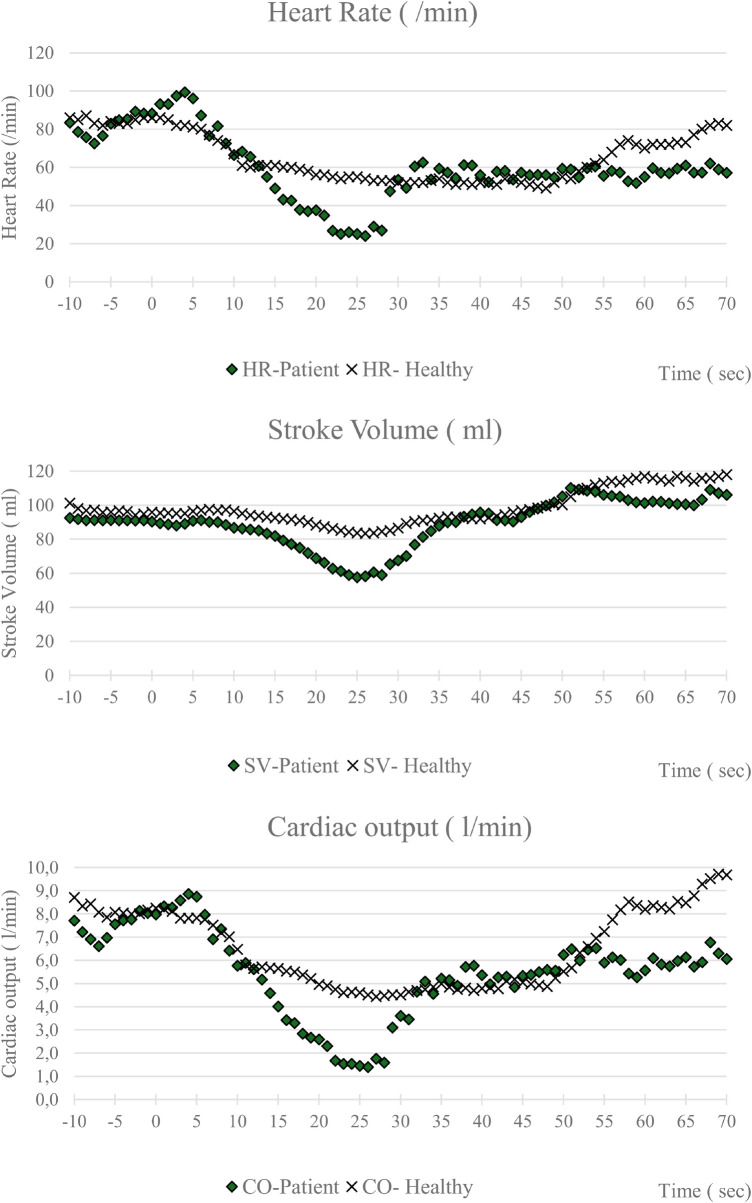
Representative example of two tests timely synchronized for clear visibility of the magnitude of the changes in heart rate, stroke volume, and cardiac output. The beginning of face water immersion starts at time 0 s. The end of the face water immersion in the patient is at 28 s and in the healthy individual at 47 s.

A representative example of two tests synchronized with the onset of face water immersion for demonstration purposes is shown in Figure 1. There was a clear difference in the magnitude of the hemodynamic changes between the two samples. The patient's heart rate did not return to baseline as a result of the development of a slow junctional rhythm, which lasted several minutes after the cessation of the test.

## 5 Discussion

In the present study, we demonstrated for the first time that athletes with a history of clinically significant symptoms while swimming also recorded a significantly stronger diving reflex, which is also considered positive from the perspective of preparticipation screening. We demonstrated that not only heart rate but also stroke volume and cardiac output were significantly more affected in the group of patients than in the group of healthy individuals. However, the underlying principle of this reaction is unclear. In addition to conventional theories surrounding dysregulation of the autonomic nervous system, our current understanding remains constrained by a dearth of comprehensive data elucidating this phenomenon, which should open the path for future studies focusing on possible connections between pathologic human diving reflex and the possible root cause of sudden cardiac death in athletes while swimming.

The underlying physiology of the diving reflex is complex and has been studied extensively (1, 7, 8). The bradycardia induced by the diving reflex was greater in trained individuals than in non-trained individuals. However, we can disregard this matter as only trained individuals were included in our study (9). The variability in HR in our study compared with the findings from the literature may depend on the participants’ position during cardiovascular measurements. For instance, in our study, participants were lying in the prone position with their faces immersed in water, whereas Smith and Kevin had participants lying supine with an ice pack on their foreheads (9). Finally, water temperature, which activates the trigeminal nerve, also plays a role in the magnitude of the diving reflex. As we used a standard protocol with the same water temperature, we can also disregard this variable (10). All hemodynamic parameters, including stroke volume and cardiac output measured in experienced breath-hold divers, declined on a scale similar to that in our group of healthy individuals (6). This implies that this variable can also be neglected. Autonomic dysfunction related to cardiovascular events is well known (11). However, current guidelines for the management of syncope do not include the possibility of testing patients with syncope via the face water immersion test for possible autonomic dysfunction (12).

Sudden cardiac death in water is a challenging and catastrophic event. Previous studies have reported conflicting results regarding the causes of these deaths. Often-mentioned pulmonary edema as the primary root cause has been challenged mainly by large cohorts of triathletes who were examined retrospectively. In a mortality autopsy study, seven out of nine deaths in water were presented with cardiovascular abnormalities during autopsy (3). The largest US Triathlon study mentioned unspecified sudden cardiac death as the primary etiology of the major root cause of death in the water (2).

In our study, the trigemino-cardiac reflex during face submersion in cold water evoked bradycardia and decreased stroke volume to the level of circulatory collapse connected to clinical symptoms. Such a response to the diving reflex has not been described earlier and is not in line with the majority of studies, which demonstrated only a mild decline in cardiac output (6). Although the diving reflex is a beneficial tool for the energy conservation principle of sudden submersion in cold water, we have demonstrated that a possible life-threatening situation is the case of overshoot, which we have seen in all of our four cases with positive clinical symptoms. The detailed underlying mechanism beyond generally known autonomic dysfunction is unfortunately unknown currently and should be a topic of further studies. The result even raises speculative question, whether the overshoot of the physiological reaction leading to the hemodynamic collapse could be a possible root cause of some of the deaths of athletes while swimming.

The major limitation of our study lies in the limited number of participants, mainly in the patient group. Due to the limited number of those cases, it is very unlikely that a single center can reach a reasonable number of participants to draw larger clinical conclusions, and a multicenter study will be needed. There are general unpublished data describing similar hemodynamic patterns in healthy individuals that we have seen in the patient group. However, we have not observed such a case in our group of healthy individuals.

## 6 Conclusions

We demonstrated that there is a statistically and clinically significant difference in hemodynamic response during the diving reflex test between groups of healthy individuals and athletes who experienced clinical symptoms when swimming. Patients with a history of clinical symptoms in the water had a bigger drop in heart rate, stroke volume, and cardiac output to the level, which might cause circulatory arrest, contrary to healthy individuals who demonstrated only mild cardio-inhibitory reactions. This novel observation of autonomic dysfunction could shed light on the possible root cause of sudden cardiac death during competitive swimming. A larger and more thorough study is needed to draw relevant clinical recommendations, as our study included a limited number of participants.

## Data Availability

The original contributions presented in the study are included in the article/Supplementary Material, further inquiries can be directed to the corresponding author.
